# Activity of Chitosan and Its Derivatives against Leishmania major and Leishmania mexicana *In Vitro*

**DOI:** 10.1128/AAC.01772-19

**Published:** 2020-02-21

**Authors:** Alaa Riezk, John G. Raynes, Vanessa Yardley, Sudaxshina Murdan, Simon L. Croft

**Affiliations:** a Department of Infection Biology, London School of Hygiene and Tropical Medicine, London, United Kingdom; b Department of Pharmaceutics, UCL School of Pharmacy, University College London, London, United Kingdom

**Keywords:** cutaneous leishmaniasis, *Leishmania major*, *Leishmania mexicana*, chitosan, macrophage uptake

## Abstract

There is an urgent need for safe, efficacious, affordable, and field-adapted drugs for the treatment of cutaneous leishmaniasis, which newly affects around 1.5 million people worldwide annually. Chitosan, a biodegradable cationic polysaccharide, has previously been reported to have antimicrobial, antileishmanial, and immunostimulatory activities. We investigated the *in vitro* activity of chitosan and several of its derivatives and showed that the pH of the culture medium plays a critical role in antileishmanial activity of chitosan against both extracellular promastigotes and intracellular amastigotes of Leishmania major and Leishmania mexicana. Chitosan and its derivatives were approximately 7 to 20 times more active at pH 6.5 than at pH 7.5, with high-molecular-weight chitosan being the most potent. High-molecular-weight chitosan stimulated the production of nitric oxide and reactive oxygen species by uninfected and *Leishmania*-infected macrophages in a time- and dose-dependent manner at pH 6.5. Despite the *in vitro* activation of bone marrow macrophages by chitosan to produce nitric oxide and reactive oxygen species, we showed that the antileishmanial activity of chitosan was not mediated by these metabolites. Finally, we showed that rhodamine-labeled chitosan is taken up by pinocytosis and accumulates in the parasitophorous vacuole of *Leishmania*-infected macrophages.

## Abstract (precis)

There is an urgent need for safe, efficacious, affordable, and field-adapted drugs for the treatment of cutaneous leishmaniasis, which newly affects around 1.5 million people worldwide annually. Chitosan, a biodegradable cationic polysaccharide, has previously been reported to have antimicrobial, antileishmanial, and immunostimulatory activities.

## INTRODUCTION

Leishmaniasis is an infectious disease caused by protozoan parasites belonging to the genus *Leishmania*. The parasite is transmitted between humans and mammalian reservoirs (e.g., dogs and rodents) through the bite of a female phlebotomine sand fly (1). There are two main clinical forms, cutaneous leishmaniasis (CL) and visceral leishmaniasis (VL), with CL being the most common (2). In addition to “simple” CL, there are other complex cutaneous manifestations, including mucocutaneous leishmaniasis (MCL), diffuse cutaneous leishmaniasis (DCL), recidivans leishmaniasis (RL), and post-kala-azar dermal leishmaniasis (PKDL) (3, 4).

CL is caused mainly by Leishmania tropica, Leishmania major, and Leishmania aethiopica in the Old World and by Leishmania braziliensis, Leishmania guyanensis, Leishmania mexicana, and Leishmania amazonensis in the New World (5). Of the 88 countries where CL occurs, 90% of the cases are in Afghanistan, Brazil, Iran, Peru, Saudi Arabia, and Syria (1). In the mammalian host, the parasite survives and multiplies within macrophages. The cellular immune responses in CL play a critical role in the control and progress of the disease and include two main mechanisms of macrophage activation: (i) the classical pathway (M1 macrophages), in which Th1 and NK cells produce cytokines (such as gamma interferon [IFN-γ]) that stimulate the production of nitric oxide (NO) and reactive oxygen species (ROS) and the activation of other lysosomal antimicrobial activities that are responsible for killing the *Leishmania* parasites, and (ii) the alternative pathway, mediated by Th2 cytokines like interleukin-4 (IL-4) and IL-13 in the early stages of infection, forming a favorable environment for *Leishmania* proliferation (6, 7).

The pentavalent antimonial compounds sodium stibogluconate (Pentostam), and meglumine antimoniate (Glucantime) have been the standard treatment for CL for the past 70 years (8). These drugs have several limitations, including difficulty of administration, toxicity of the drug, and varying sensitivities among *Leishmania* species (9). Second-line treatments include the polyene antifungal amphotericin B, which also suffers from toxicity, the oral phospholipid miltefosine, the use of which is limited by teratogenicity, and the aminoglycoside antibiotic paromomycin (PM), which has low cure rates for certain *Leishmania* species (10–12). Treatment with intravenous liposomal amphotericin B (AmBisome) is safe and has achieved clinical success against CL at a dose of 3 mg/kg of body weight daily for 7 days (13, 14), but the high cost of this formulation limits its use (15). Two Cochrane analyses have clearly shown clinical deficiencies of most drugs (16, 17). There is an urgent need for new treatments which can eliminate the parasites and improve the healing process and are safe, reliable, and also field adaptable for use in diverse health care systems.

Chitosan is a biodegradable, biocompatible, positively charged nontoxic mucoadhesive biopolymer produced by the deacetylation of chitin. Chitosan has a pK_a_ of approximately 6.3 and is insoluble at alkaline pH but soluble in weak acidic solvents like acetic acid, where the amino groups become protonated. Many reports have described the antimicrobial activity of chitosan, but the actual mechanism of action has not been fully elucidated (18), although three direct mechanisms have been suggested. The first is the interaction between the protonated NH_3_^+^ groups of chitosan and the negatively charged cell membrane of microbes. This interaction changes the permeability of the microbial cell membrane, causing osmotic imbalances and consequently killing the microbe (18, 19). The second suggested mechanism is that chitosan binds to microbial DNA and inhibits DNA transcription, assuming that chitosan penetrates the microbial cell membrane and reaches the DNA (19, 20). The third mechanism is via chitosan’s chelation of metals and binding of basic nutrients essential for microbial growth (19). An indirect mechanism of action may be related to the known proinflammatory effect of chitosan on macrophages. This involves stimulation of tumor necrosis factor alpha (TNF-α), IL-6, NO, ROS, and IFN-γ, which play critical roles in the proinflammatory response against intracellular microbes by enhancing the production of microbicidal reactive nitrogen species (21–25). Chitosan activates polymorphonuclear leukocytes, macrophages, and fibroblasts, and these properties promote wound healing (18, 26).

The poor solubility of chitosan and the loss of the cationic charge in neutral and alkaline environments are two of the major obstacles to the consideration of chitosan as a useful antimicrobial. Recently, the chemical modification of chitosan to produce various derivatives to improve its solubility and widen its application has gained attention (27, 28). Chitosan and its derivatives have been shown to have *in vitro* antileishmanial activity with 50% effective concentrations (EC_50_s) ranging from 70 to 240 μg/ml against L. infantum, L. amazonensis, and Leishmania chagasi promastigotes and amastigotes (29–34). All this makes chitosan an appropriate candidate for further studies to evaluate its suitability for the treatment of CL.

The aim of our work was to (i) determine the *in vitro* antileishmanial activity of chitosan and its derivatives against L. major and L. mexicana promastigotes and intracellular amastigotes at two different pHs (the culture medium pH of 7.5 and a lower pH of 6.5, which are both suitable for macrophage and parasite growth) (35–37), (ii) evaluate the *in vitro* role of chitosan in the activation of the macrophage M1 proinflammatory phenotype via the measurement of NO, ROS, and TNF-α production by host cells and by measuring parasite survival, and (iii) investigate chitosan uptake by macrophages to explain its activity against intracellular amastigotes.

## RESULTS

### *In vitro* activities of chitosan and derivatives against L. major and L. mexicana.

The antileishmanial activities (against promastigotes and amastigotes) of high-, medium-, and low-molecular-weight (HMW, MMW, and LMW, respectively) chitosan and its derivatives (a total of 11 compounds) were tested. Dose-dependent activity (Fig. S1 and S2 in the supplemental material) against *Leishmania* promastigotes and amastigotes was observed for chitosan and its derivatives, except for carboxymethyl chitosan, which showed no activity against either parasite stage within the experimental parameters tested (pH 7.5 or 6.5 and concentrations up to 400 μg/ml). In the 72-h assays, chitosan and its derivatives (except for carboxymethyl chitosan) were 7 to 20 times more active against L. major and L. mexicana promastigotes and intracellular amastigotes (infecting peritoneal mouse macrophages [PEMs]) in culture medium at pH 6.5 than at pH 7.5 (*P* < 0.05 by *t* test) (Tables 1 and 2). HMW, MMW, and LMW chitosan, from both crustacean and fungal sources, exhibited significantly higher activities than chitosan derivatives against promastigotes and intracellular amastigotes (EC_50_s of ≈6 μg/ml against L. major promastigotes and 10 μg/ml against L. mexicana promastigotes, and EC_50_s of ≈12 μg/ml against L. major amastigotes and 16 μg/ml against L. mexicana amastigotes) at pH 6.5 (*P* < 0.05 by an extra-sum-of-squares F test) (Tables 1 and 2). Additionally, L. major promastigotes and amastigotes were significantly more sensitive to chitosan and its derivatives than L. mexicana promastigotes and amastigotes (approximately 1.5 to 2 times more sensitive [*P* < 0.05 by the extra-sum-of-squares F test]).

**TABLE 1:** *In vitro* activities of chitosan and its derivatives against promastigotes at two pHs

Compound	Mean value ± SD (μg/ml) at^*a*^:							
	pH 7.5				pH 6.5^*b*^			
	L. major		L. mexicana		L. major		L. mexicana	
	EC_50_	EC_90_	EC_50_	EC_90_	EC_50_	EC_90_	EC_50_	EC_90_
Amphotericin B	0.05 ± 0.01	0.2 ± 0.02	0.14 ± 0.01	0.3 ± 0.03	0.07 ± 0.02	0.3 ± 0.1	0.13 ± 0.07	0.3 ± 0.02
HMW chitosan	105 ± 12	1,549 ± 525	140 ± 12	2,187 ± 928	5.9 ± 0.5	37 ± 9	10.4 ± 1.6	98 ± 33
MMW chitosan	113 ± 9	1,277 ± 580	150 ± 12	2,223 ± 681	6.2 ± 0.3	43 ± 8	10.9 ± 1.4	96 ± 27
LMW chitosan	118 ± 11	1,238 ± 582	157 ± 13	2,225 ± 723	6.7 ± 0.3	40 ± 8	10.2 ± 1.5	84 ± 28
Fungal chitosan	118 ± 11	1,228 ± 560	150 ± 13	1,991 ± 580	6.2 ± 0.3	42 ± 6	10.5 ± 1.3	61 ± 17
Chitosan oligosaccharide	153 ± 15	1,680 ± 506	190 ± 20	2,366 ± 461	62.5 ± 4	446 ± 92	77 ± 2.7	452 ± 36
Chitosan oligosaccharide-lactate	98 ± 9	1,226 ± 130	125 ± 14	765 ± 83	14 ± 0.1	135 ± 2	23 ± 1.4	311 ± 25
Chitosan HCl	96 ± 7	1,189 ± 211	110 ± 24	746 ± 169	13.2 ± 1	118 ± 34	20.8 ± 2.4	264 ± 61
PC1-chitosan^*c*^	111 ± 20	1,875 ± 230	176 ± 14	2,832 ± 412	19.9 ± 2.8	187 ± 90	32 ± 2.2	328 ± 48
PC2-chitosan^*c*^	104 ± 6	1,485 ± 259	170 ± 8	2,744 ± 377	16.5 ± 2.7	138 ± 49	28 ± 2.4	296 ± 53
PC3-chitosan^*c*^	119 ± 19	1,860 ± 365	187 ± 16	3,175 ± 580	23.3 ± 2.5	218 ± 44	37 ± 2.5	442 ± 65
Carboxymethyl chitosan	—^*d*^	—	—	—	—	—	—	—

a Experiments were conducted in triplicate cultures. Experiments were reproduced a further two times with confirmed similar data (not shown). Amphotericin B deoxycholate was used as a positive control. Both RPMI alone at pH 6.5 and chitosan solvent did not show any activity against promastigotes. Statistically significant differences were found for the EC_50_s of chitosan and its derivatives at pH 6.5 and pH 7.5 (*P* < 0.05 by using *t* test).

b L. major promastigotes were significantly more susceptible to chitosan and derivatives than L. mexicana at pH 6.5 (*P* < 0.05 by the extra-sum-of-squares F test).

c Phosphorylcholine-substituted chitosan (see Table 4).

d —, no activity up to 400 μg/ml.

**TABLE 2:** *In vitro* activities of chitosan and its derivatives against amastigotes infecting PEMs, as well as cytotoxicities toward KB cells

Compound	Mean value ± SD (μg/ml)^*a*^									
	Activity against amastigotes at^*b*^:								Cytotoxicity toward KB cells at pH 6.5^*c*^	
	pH 7.5				pH 6.5					
	L. major		L. mexicana		L. major		L. mexicana			
	EC_50_	EC_90_	EC_50_	EC_90_	EC_50_	EC_90_	EC_50_	EC_90_	LD_50_	LD_90_
Amphotericin B	0.07 ± 0.01	0.13 ± 0.05	0.19 ± 0.05	1.5 ± 0.2	0.06 ± 0.01	0.11 ± 0.06	0.18 ± 0.06	1.7 ± 0.3	58 ± 8	190 ± 9
HMW chitosan	98 ± 6	1,635 ± 245	119 ± 9	1,804 ± 304	11.4 ± 1	69 ± 18	15.4 ± 2	103 ± 28	752 ± 90	3,022 ± 366
MMW chitosan	103 ± 8	1,652 ± 287	125 ± 10	1,793 ± 323	12.9 ± 1	81 ± 18	16.3 ± 2	122 ± 34	758 ± 89	3,019 ± 400
LMW chitosan	102 ± 7	1,651 ± 282	125 ± 10	1,795 ± 320	12.1 ± 1	74 ± 14	16.1 ± 2	116.6 ± 33	803 ± 90	3,088 ± 420
Fungal chitosan	102 ± 7	1,650 ± 276	124 ± 9	1,796 ± 316	12.6 ± 3	92 ± 27	16.9 ± 2	144 ± 44	759 ± 91	3,134 ± 380
Chitosan oligosaccharide	145 ± 12	2,473 ± 500	175 ± 14	2,543 ± 505	73 ± 4	260 ± 32	86.2 ± 6	288 ± 39	765 ± 93	3,232 ± 400
Chitosan oligosaccharide-lactate	93 ± 7	1,957 ± 174	120 ± 9	2,365 ± 239	39 ± 1	201 ± 16	47 ± 2	245 ± 23	754 ± 92	3,058 ± 390
Chitosan HCl	97 ± 11	2,080 ± 516	121 ± 15	2,402 ± 667	40 ± 2	210 ± 23	47.9 ± 3	243 ± 33	781 ± 92	3,589 ± 405
PC1-chitosan^*d*^	144 ± 10	1,292 ± 217	169 ± 12	1,365 ± 212	68 ± 3	246 ± 26	81.7 ± 6	274 ± 38	756 ± 93	3,364 ± 398
PC2-chitosan^*d*^	133 ± 6	1,005 ± 194	159 ± 6	1,705 ± 170	60 ± 3	202 ± 22	71.9 ± 5	237 ± 36	800 ± 92	3,709 ± 410
PC3-chitosan^*d*^	163 ± 11	1,052 ± 144	187 ± 10	1,107 ± 142	71 ± 4	251 ± 30	83.5 ± 6	286 ± 41	786 ± 93	3,719 ± 378
Carboxymethyl chitosan	—^*e*^	—	—	—	—	—	—	—	1,184 ± 99	3,999 ± 500

a Experiments were conducted in quadruplicate cultures. Experiments were reproduced a further two times with similar results (data not shown).

b Both RPMI alone at pH 6.5 and chitosan solvent did not show any activity against amastigotes. Statistically significant differences were found between the EC_50_s of chitosan and its derivatives at pH 6.5 and pH 7.5 (*P* < 0.05 by using *t* test).

c Chitosan and its derivatives had low cytotoxicities toward KB cells at both pH 6.5 and 7.5, and there was no significant difference in the cytotoxicities at these two pHs (*P* < 0.05 by *t* test). No statistically significant difference was found between LD_50_s (50% lethal dose) of the three types of chitosan and other derivatives against KB cells (except for carboxymethyl chitosan, which was the least toxic) (*P* > 0.05 by the extra-sum-of-squares F test).

d Phosphorylcholine-substituted chitosan (see Table 4).

e —, no activity up to 400 μg/ml.

To allow like-for-like comparison, EC_50_s were recalculated in terms of molarity, using estimated molecular weights (HMW chitosan, 342.5 kDa; MMW chitosan, 250 kDa; LMW chitosan, 120 kDa, and fungal chitosan, 130 kDa) at pH 6.5. Based on molarity (Tables S4 and S5), HMW chitosan was significantly more active against L. major and L. mexicana promastigotes and amastigotes and, hence, was used in all subsequent studies.

### Host cell dependence of the antileishmanial activity of HMW chitosan at pH 6.5.

We aimed to assess the host cell dependence of the antileishmanial activity of HMW chitosan and amphotericin B (Fungizone) by evaluating the *in vitro* activity against L. major amastigotes in three different macrophage types; the EC_50_s and EC_90_s in the three different macrophage populations are summarized in Table 3. There was a significant difference in the activity of HMW chitosan depending on the type of macrophage (PEMs, bone marrow-derived macrophages [BMMs], or human leukemic monocyte-like-derived cells [THP-1 cell line]) (*P* < 0.05 by the extra-sum-of-squares F test). HMW chitosan was significantly more active against intracellular amastigotes in PEMs and BMMs than in differentiated THP-1 cells.

**TABLE 3:** HMW chitosan activities against L. major amastigotes in three different macrophage cultures

Host cells	Mean value ± SD^*a*^			
	HMW chitosan (μg/ml)		Amphotericin B (μM)	
	EC_50_	EC_90_	EC_50_	EC_90_
PEMs	10.31 ± 1.22	89.07 ± 20.46	0.02 ± 0.004	0.27 ± 0.07
BMMs	14.60 ± 1.79	145.7 ± 36.2	0.04 ± 0.005	0.43 ± 0.1
THP-1	24.28 ± 2.87	200.1 ± 48.8	0.08 ± 0.006	1.15 ± 0.37

a More than 80% of macrophages were infected at 24 h for all cell lines. Cultures were grown at pH 6.5 and analyzed after 72 h. Experiment was conducted in quadruplicate cultures. Experiment was reproduced a further two times with similar results (data not shown). There were statistically significant differences in EC_50_s between the three types of cells (*P* < 0.05 by the extra-sum-of-squares F test); chitosan and amphotericin B were significantly more active in PEMs and BMMs than in THP-1 cells. RPMI and DMEM alone at pH 6.5 and chitosan solvent did not show any activity against amastigotes.

### Effects of HMW chitosan on the production of TNF-α by uninfected or L. major-infected BMMs at pH 6.5.

**FIG 1:**
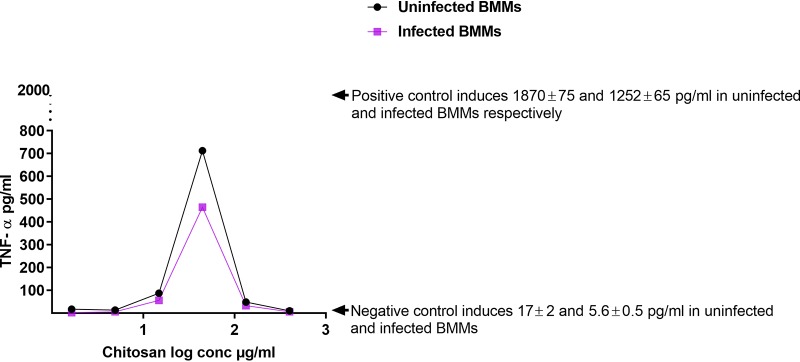
TNF-α production in uninfected and L. major-infected BMMs after 24 h of exposure to 1.64, 4.9,14.8, 44.4, 133.3, or 400 μg/ml of chitosan at pH 6.5. The dose response in both uninfected and L. major-infected BMMs was bell shaped. TNF-α production was significantly decreased (*P* < 0.05 by *t* test) by infecting the cells with L. major. Experiment was conducted in quadruplicate, and data are expressed as mean values ± SD. Experiment was reproduced a further two times with similar results (data not shown). Positive control was BMMs treated with LPS at 10 μg/ml. Negative control was BMMs not exposed to chitosan. Initial macrophage infection rate was >80% after 24 h. Chitosan solvent did not cause any TNF-α production.

The activation of M1 macrophages by Th1 lymphocytes plays an important role in the control of CL (6, 38, 39). Therefore, we measured TNF-α production by BMMs stimulated by HMW chitosan. Following exposure to HMW chitosan, the TNF-α production by BMMs was found to be dose dependent, in a bell-shaped manner, in both *Leishmania*-infected and uninfected cells, as shown in Fig. 1. After 24 h, the levels of TNF-α in the culture fluid of BMMs exposed to HMW chitosan at concentrations of 14.8, 44.4, and 133.3 μg/ml were significantly higher than the levels in culture fluid of BMMs (infected and uninfected) that had not been exposed to chitosan, with TNF-α being the highest at 44.4 μg/ml chitosan. Meanwhile, at other concentrations (1.64, 4.9, and 400 μg/ml), HMW chitosan did not stimulate BMMs to produce TNF-α (*P* < 0.05 by *t* test).

HMW chitosan at concentrations of 14.8, 44.4, and 133.3 μg/ml stimulated BMMs to produce TNF-α at concentrations of 87 ± 4.5 (mean ± standard deviation [SD]), 712 ± 9, and 48 ± 3 pg/ml, respectively, in uninfected BMMs and 56 ± 3.5, 464 ± 10, and 32 ± 4 pg/ml, respectively, in L. major-infected BMMs. Less TNF-α was generated when the chitosan concentration was increased to 133.3 μg/ml and above. Lipopolysaccharides (LPS) from Escherichia coli O26:B6 (positive control) stimulated TNF-α production in both uninfected and infected BMMs after a 24-h incubation period at a significantly higher level than did chitosan (*P* < 0.05 by *t* test). Our results indicated that HMW chitosan activated M1 macrophages.

### Effects of HMW chitosan on the production of ROS by BMMs at pH 6.5.

ROS plays an important role in the killing of intracellular amastigotes (6, 38, 39), and therefore, we measured ROS production by BMMs stimulated by HMW chitosan. HMW chitosan (at concentrations of 14.8, 44.4, and 133.3 μg/ml) increased the production of ROS (indicated by H_2_DCFDA [2′,7′-dichlorodihydrofluorescein diacetate] fluorescence) after 4 h of incubation but did not stimulate ROS after 8 h of incubation (Table S1). Other concentrations of HMW chitosan (1.64, 4.9, and 400 μg/ml) did not stimulate BMMs to produce ROS after 4 h or 8 h of incubation.

**FIG 2:**
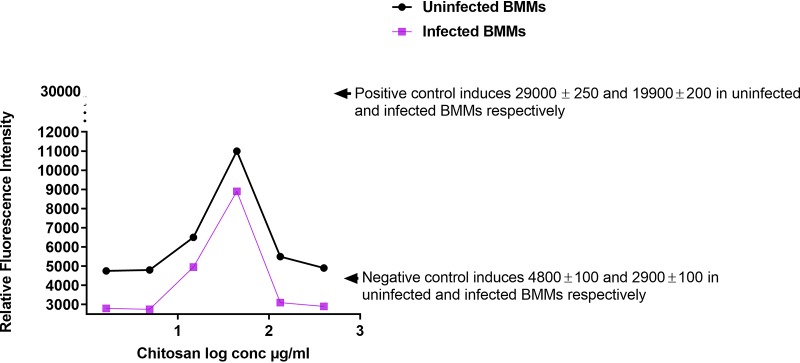
ROS production in uninfected and L. major-infected BMMs after 4 h of exposure to 1.64, 4.9,14.8, 44.4, 133.3, or 400 μg/ml of HMW chitosan at pH 6.5. High levels of ROS were induced by both uninfected and L. major-infected BMMs exposed to HMW chitosan compared to the levels in BMMs that were not exposed to chitosan (*P* < 0.05 by *t* test). Maximum production of ROS occurred at 44.4 μg/ml of chitosan. ROS production by L. major-infected BMMs was significantly lower than in uninfected cells (*P* < 0.05 by *t* test). Experiment was conducted in quadruplicate, and data are expressed as mean values ± SD. Experiment was reproduced a further two times with similar results (data not shown). Positive control was BMMs treated with 25 mM H_2_O_2_ (a known ROS inducer). Negative control was BMMs not exposed to chitosan. Initial macrophage infection rate was >80% after 24 h. Chitosan solvent alone did not cause any ROS production.

The ROS dose response in both uninfected and infected BMMs was bell shaped, similar to that seen with TNF-α. Increasing the chitosan concentration from 14.8 to 44.4 μg/ml increased ROS production, after which further increases in concentration reduced ROS production. In addition, ROS production by BMMs was significantly decreased (*P* < 0.05 by *t* test) by infecting the cells with L. major, as shown by the results in Fig. 2.

**FIG 3:**
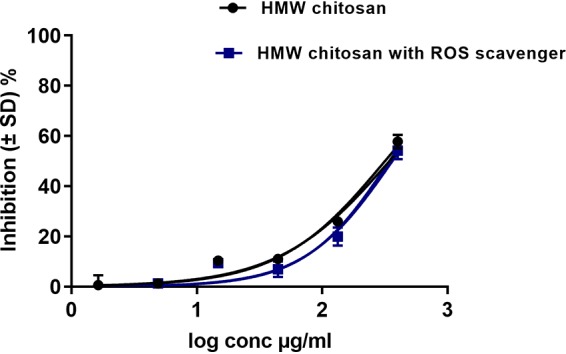
Activity of HMW chitosan against L. major amastigotes in BMMs after 4 h, with and without ROS scavenger at pH 6.5. Infected macrophages were preincubated with 5 mM NAC for 2 h, after which HMW chitosan at a concentration of 1.64, 4.9,14.8, 44.4, 133.3, or 400 μg/ml was added and the cells were incubated for a further 4 h. Chitosan activity against intracellular amastigotes was evaluated as described in Materials and Methods. Values are expressed as percentages of inhibition of infection relative to the results for untreated controls. After 4 h, there was no significant difference in the antileishmanial activities of chitosan after scavenging of ROS (*P* > 0.05 by *t* test). Experiment was conducted in quadruplicate, and data are expressed as mean values ± SD. Experiment was reproduced a further two times with similar results (data not shown). Initial macrophage infection rate was >80% after 24 h.

We found that *in vitro*, HMW chitosan had a stimulatory effect on BMM ROS production after 4 h of incubation. We therefore investigated whether this ROS plays any role in the activity of HMW chitosan against intracellular amastigotes. For these experiments, the 4-h-posttreatment time point was taken because ROS peaked at this point in BMMs in response to chitosan treatment, at a time when chitosan does not induce NO in BMMs (Table S3). Scavenging of ROS by the ROS scavenger 5 mM *N*-acetyl-l-cysteine (NAC) had no significant impact on the activity of chitosan against intracellular amastigotes (*P* > 0.05 by *t* test) (Fig. 3). The ROS scavenger caused complete scavenging of ROS production after 4 h (Table S2) and had no cytotoxicity against human squamous carcinoma cells (KB cells) or leishmanicidal activity against L. major amastigotes (data not shown). Even though chitosan stimulated ROS production, ROS did not play a role in the antileishmanial activity of chitosan.

### Effects of HMW chitosan on the production of NO by BMMs at pH 6.5.

**FIG 4:**
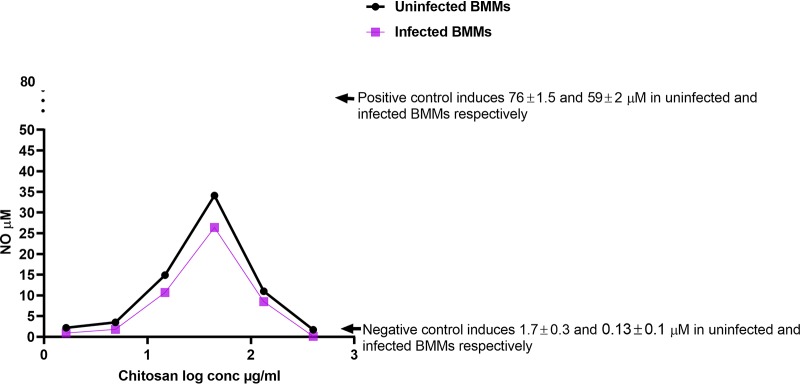
NO production in uninfected and L. major-infected BMMs after 24 h of exposure to 1.64, 4.9,14.8, 44.4, 133.3, or 400 μg/ml of chitosan at pH 6.5. The response in both uninfected and infected BMMS was bell shaped and related to chitosan concentration. Maximal production of NO was stimulated by 44.4 μg/ml of chitosan. NO production was significantly decreased (*P* < 0.05 by *t* test) when the cells had been infected with L. major. Experiment was conducted in quadruplicate cultures, and data are expressed as mean values ± SD. Experiment was reproduced a further two times with similar results (data not shown). Positive control was BMMs treated with 10 μg/ml LPS. Negative control was BMMs not exposed to chitosan. Initial macrophage infection rate was >80% after 24 h. Chitosan solvent alone did not cause any NO production.

NO plays an important role in the killing of intracellular amastigotes (6, 38, 39), and therefore, we measured NO production by BMMs stimulated by HMW chitosan. We showed that chitosan did not have a stimulatory effect on BMM NO production after 4 h of incubation (Table S3). However, after 24 h of incubation, HMW chitosan had a stimulatory effect on BMM NO production at pH 6.5 in a clearly bell-shaped, dose-dependent manner (Fig. 4). HMW chitosan at concentrations of 14.8, 44.4, and 133.3 μg/ml induced both uninfected and infected BMMs to produce NO (at 14.9 ± 0.3, 34 ± 1.2, and 11 ± 1 μM, respectively, in uninfected BMMs and 11 ± 1, 26 ± 2.5 and 8 ± 1.2 μM, respectively, in infected BMMs), NO being highest at 44.4 μg/ml. Meanwhile, other concentrations of HMW chitosan (1.64, 4.9, and 400 μg/ml) did not stimulate BMMs to produce NO after 24 h of incubation.

LPS caused significantly higher NO production than did HMW chitosan (*P* < 0.05 by *t* test) in both uninfected and infected BMMs. The levels of NO produced by L. major-infected BMMs exposed to LPS (positive control) or HMW chitosan were significantly lower than the levels produced by uninfected BMMs (*P* < 0.05 by *t* test) (Fig. 4).

**FIG 5:**
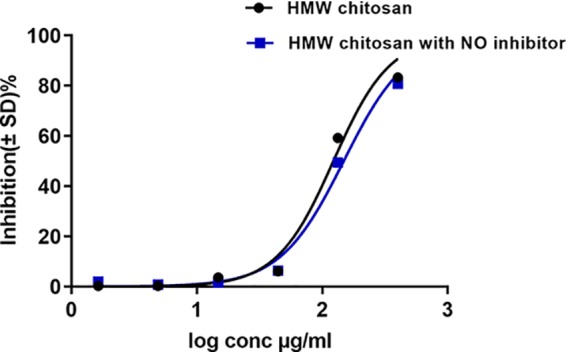
Activity of HMW chitosan against L. major-infected BMMs after 24 h in the presence or absence of an NO inhibitor at pH 6.5. Infected macrophages were preincubated with the NO inhibitor L-NMMA (0.4 mM) for 2 h, following which HMW chitosan at a concentration of 1.64, 4.9,14.8, 44.4, 133.3, or 400 μg/ml was added and the cells were incubated for a further 24 h. Chitosan activity against intracellular amastigotes was evaluated as described in Materials and Methods. Values are expressed as percentages of inhibition of infection relative to the results for untreated controls. After 24 h, there was no significant difference in the activity of chitosan after inhibition of NO (*P* > 0.05 by *t* test). Experiment was conducted in quadruplicate cultures, and data are expressed as mean values ± SD. Experiment was reproduced a further two times and the results were confirmed (data not shown). Initial macrophage infection rate was >80% after 24 h.

As HMW chitosan had an *in vitro* stimulatory effect on BMM NO production after 24 h of incubation, we investigated further whether NO has any role in the activity of HMW chitosan against intracellular amastigotes. Inhibition of NO production by the NO inhibitor N^G^-methyl-l-arginine acetate salt (L-NMMA) at 0.4 mM had no significant influence on the activity of chitosan against intracellular amastigotes (*P* > 0.05 by *t* test) (Fig. 5), although the NO inhibitor did cause a complete inhibition of NO production (Table S2) after 24 h and had no cytotoxic effects against KB cells and no leishmanicidal activity against intracellular L. major amastigotes (data not shown). Even though chitosan stimulated NO production, it did not play a role in the antileishmanial activity of chitosan.

### Cellular uptake of HMW chitosan and inhibition of endocytosis.

**FIG 6:**
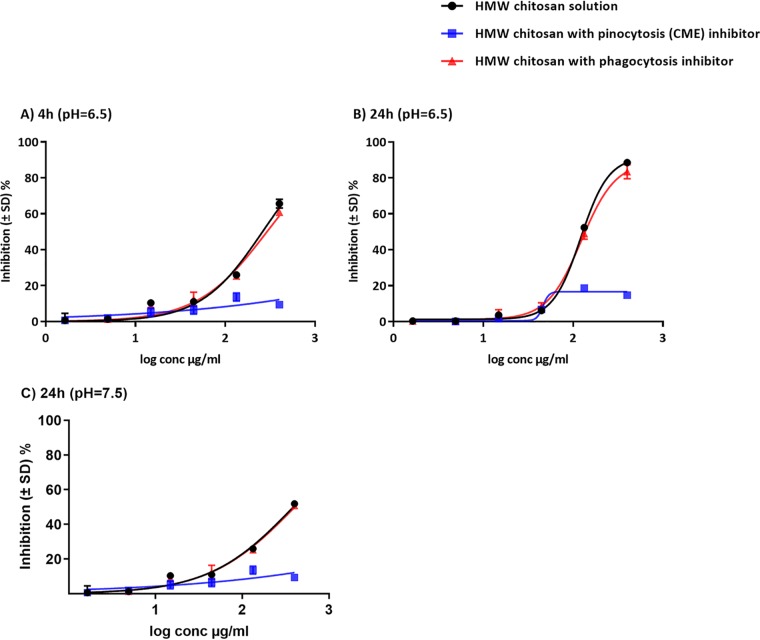
Activities of HMW chitosan against L. major-infected BMMs after 4 h at pH 6.5 (A), 24 h at pH 6.5 (B), or 24 h at pH 7.5 (C), with or without phagocytosis inhibitor or pinocytosis (CME) inhibitor. We found that chitosan requires pinocytosis (CME) and not phagocytosis by BMMs for killing of L. major amastigotes at pH 6.5 and 7.5. BMMs were infected with stationary-phase promastigotes. Some of the infected macrophages were preincubated with cytochalasin D (phagocytosis inhibitor) or dynasore (pinocytosis [CME] inhibitor) and exposed to various concentrations (1.64, 4.9,14.8, 44.4, 133.3, or 400 μg/ml) of chitosan for 4 h and 24 h, followed by microscopic counting of the number of infected macrophages. There was no significant difference in the activities of HMW chitosan after inhibition of phagocytosis (*P* > 0.05 by *t* test). In contrast, significant inhibition of chitosan-mediated parasite killing occurred in the presence of dynasore at the two pH values (*P* < 0.05 by *t* test). Values are expressed as percentages of inhibition of infection relative to the results for untreated controls. Experiments were conducted in quadruplicate cultures, and data are expressed as mean values ± SD. Experiments were reproduced a further two times and the results confirmed (data not shown). Initial macrophage infection rate was >80% after 24 h.

We found that the activation of M1 macrophages by HMW chitosan did not play a role in its activity against intracellular amastigotes. Therefore, we investigated whether the antileishmanial effects of HMW chitosan against intracellular amastigotes after 4 h and 24 h of exposure were dependent on the direct activity of chitosan following its entry into the macrophages at pH 6.5. No significant difference was observed in the activity of chitosan against intracellular amastigotes when it was added after prior phagocytosis inhibition with cytochalasin D (*P* > 0.05 by *t* test) (Fig. 6). In contrast, dynasore, an inhibitor of pinocytosis (clathrin-mediated endocytosis [CME]), did significantly affect chitosan-mediated parasite killing at pH 6.5 (*P* < 0.05 by *t* test) (Fig. 6). The same activity was seen at pH 7.5 (Fig. 6C). The two inhibitors had no cytotoxicity against KB cells or activity against intracellular L. major amastigotes at the concentrations used. Pinocytosis (CME) played a critical role in the efficacy of HMW chitosan against intracellular amastigotes.

### Fluorescence microscopy of the uptake of chitosan by macrophages.

**FIG 7:**
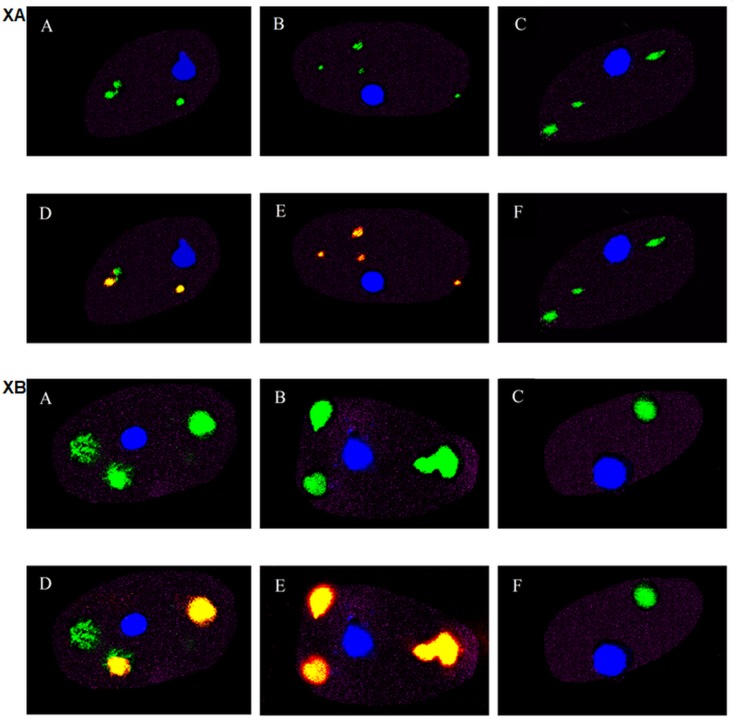
Fluorescence microscopy images of the cellular uptake of rhodamine-labeled chitosan at 4 h and 24 h at pH 6.5 by BMMs infected with L. major-GFP (XA) or with L. mexicana-GFP (XB). Blue represents the nuclei of BMMs, green represents intracellular amastigotes, red represents labeled chitosan, and yellow represents merged red chitosan and green *Leishmania* parasites. (A to F) Infected BMMs unexposed to chitosan after 4 h (A) or 24 h (B), exposed to chitosan after 4 h (D) or 24 h (E), unexposed to chitosan after 24 h (C), and exposed to chitosan and pinocytosis inhibitor (dynasore) after 24 h (F).

Rhodamine-labeled chitosan was used to track the delivery of chitosan to the parasitophorous vacuoles (PVs) of *Leishmania*-infected macrophages. Fig. 7 illustrates the cellular uptake of chitosan by BMMs infected with green fluorescent protein (GFP)-labeled L. major (L. major-GFP) or L. mexicana-GFP after 4 h and 24 h of exposure to rhodamine-labeled chitosan. There was colocalization of chitosan and intracellular amastigotes after 4 h and 24 h, with normalized mean deviation product (nMDP) color indexes of 0.7 and 1, respectively (see “Microscopic imaging of the cellular uptake of rhodamine-labeled chitosan” in Materials and Methods). The uptake of chitosan increased in a time-dependent manner. The results in Fig. 7XA and XB, panels D and E, show this uptake after 4 h and 24 h, respectively, as well as the accumulation of chitosan in PVs (Fig. 7, colocalization of rhodamine and GFP shown by yellow color). The results in Fig. 7XA and XB, panels F, also show that the inhibition of pinocytosis (CME) with dynasore prevented the uptake of chitosan, with a negative nMDP color index that represents no colocalization of chitosan and amastigotes. This is also supporting evidence for the uptake by pinocytosis as seen by the results in Fig. 6.

## DISCUSSION

The literature on the antileishmanial activity of chitosan and its derivatives is limited, especially pertaining to its mechanism(s) of action (19, 40, 41). In this study, we assessed the antileishmanial activity of various forms of chitosan, including low-, medium-, and high-molecular-weight chitosan and chitosan derivatives. Chitosan derivatives are generally produced by chemical modification of the amino or hydroxyl groups of chitosan for the optimization of the physicochemical properties. We found that chitosan and its derivatives had minimal cytotoxicity against KB cells, with 50% lethal doses (LD_50_s) of ≥750 μg/ml in RPMI 1640 at pH 7.5 or 6.5. These data support previous reports of chitosan’s low cytotoxicity against CCRF-CEM (human lymphoblastic leukemia) and L132 (human embryonic lung) cells that had similar LD_50_s (42, 43).

We determined that a lower pH of 6.5 enhanced the antileishmanial activities of chitosan and its derivatives against L. major and L. mexicana promastigotes and amastigotes by 7 to 20 times compared to the results at pH 7.5. This higher activity of chitosan at the lower pH of 6.5 could be due to its greater ionization (protonation of the amino groups; the pK_a_ of chitosan is ≈6.3). The greater positive charge could increase the antimicrobial activity of chitosan due to interaction with the negatively charged microbial membrane (in accordance with the first postulated mechanism of antimicrobial activity described in the introduction) (18, 19). A higher chitosan activity at lower pH (pH ≈5) has previously been reported against Escherichia coli and Salmonella enterica serovar Typhimurium (44, 45).

**FIG 8:**
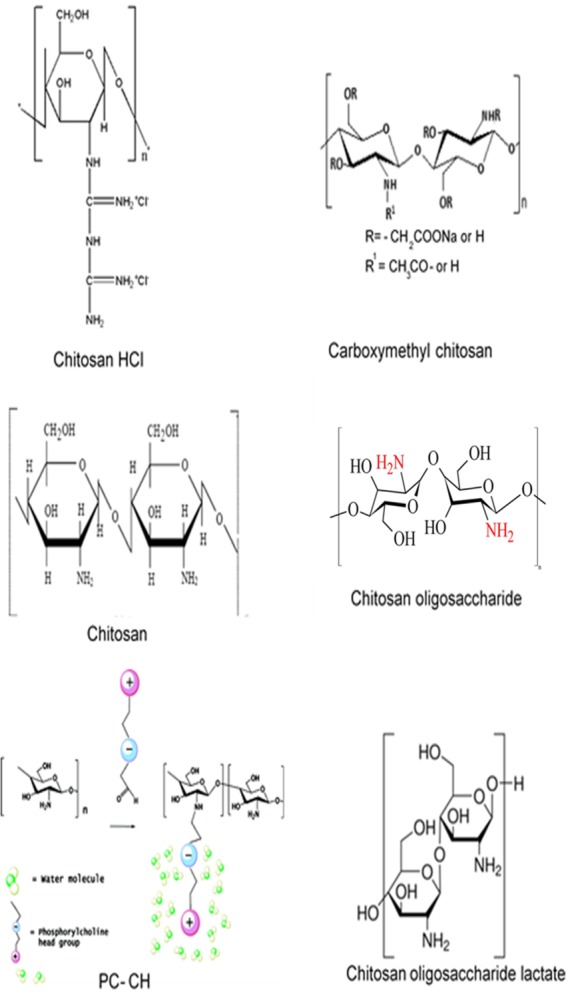
The structures of chitosan (60) and its derivatives, chitosan HCl, carboxymethyl chitosan (61), chitosan oligosaccharide (60), PC-chitosan (republished from reference 28 with permission of the publisher), and chitosan oligosaccharide lactate (59).

Our study is the first to show the pH dependence of the antileishmanial activity of chitosan and its derivatives and could explain why reports of the antileishmanial activity of chitosan in the literature have shown such variability, with EC_50_s ranging from 70 to 240 μg/ml against L. infantum, L. amazonensis, and L. chagasi promastigotes and amastigotes (29–34). For example, in one study, the EC_50_ of chitosan against L. infantum amastigotes (in PEMs) in RPMI 1640 medium was 100.81 μg/ml, but the pH at which the experiment was conducted was not mentioned (29). The influence of pH was also seen when the antileishmanial activities of chitosan (of the different molecular weights) and chitosan derivatives were compared. While the different chitosans and derivatives showed minor differences in their antileishmanial activities at pH 7.5, the derivatives were 3 to 5 times less active than the HMW, MMW, LMW, and fungal chitosan at the lower pH of 6.5. This reduced activity could be due to the smaller number of amino groups on the chitosan derivatives (see Fig. 8). These derivatives are more soluble at a higher pH and have activities similar to those of chitosan, but at a lower pH, the higher protonation of the chitosan improves the antileishmanial activity significantly (46, 47). Carboxymethyl chitosan had no antileishmanial activity—most of the amino groups on this derivative have been replaced by carboxymethyl moieties, making the molecule negatively charged (48).

The higher antileishmanial activity of HMW chitosan compared to the activities of MMW and LMW chitosan mirrors its greater antibacterial activity in another study against Escherichia coli, Pseudomonas aeruginosa, and Staphylococcus aureus (49). HMW has a long chain and, therefore, more glucosamine units and possesses more amino groups (Fig. 8), resulting in more protonated groups (-NH_3_^+^) than MMW and LMW chitosan have (49), which could explain its greater potency.

We also showed that the antileishmanial activity of chitosan is significantly greater against L. major-infected PEMs or BMMs than against L. major-infected differentiated THP-1 cells, in the order PEMs > BMMs > THP-1 cells, underlining the need to take the host cell into consideration when conducting similar experiments (50).

In order to understand the potential antiamastigote mechanism(s) of chitosan, we investigated whether the activity of HMW chitosan against the intracellular amastigotes was via direct uptake into the host cell and localization in the parasitophorous vacuole or indirectly via the activation of M1 macrophages, given that the cellular immune responses in cutaneous leishmaniasis play a critical role in self-cure (51, 52).

The activation of M1 macrophages by the Th1 lymphocyte subpopulation, which produces different cytokines, primarily IFN-γ and TNF-α, is crucial for the killing of intracellular *Leishmania* via the triggering of an oxidative burst, and therefore, the host cells increase the production of ROS and NO, which are responsible for killing of the parasite (38, 39). We found that HMW chitosan stimulated TNF-α production by macrophages, and this would be expected to be an indicator of an M1 macrophage that would have greater leishmanicidal activity. Our results show that chitosan stimulated BMM ROS production with a peak after 4 h and led to significant increases in the TNF-α and NO production after 24 h in a bell-shaped response. Similar findings have been reported showing that HMW chitosan had an *in vitro* stimulatory effect on NO production in PEMs (from male rats) (25) and LMW chitosan stimulated RAW264.7 macrophage TNF-α production (24). Another study demonstrated that LMW chitosan induced ROS production in an epithelial, human breast cancer cell line (53). The bell-shaped responses are consistent with a study that showed that chitosan stimulated NO and TNF-α production in peritoneal macrophages in a dose-dependent manner and that their levels tended to decrease at higher concentrations of chitosan (320 μg/ml) (54). This type of response has also been reported previously for tucaresol for both its immunomodulatory activity and activity against experimental Leishmania donovani infections, albeit at lower doses (55). Despite the observed chitosan-induced ROS and NO production, there was no evidence that this contributed to the antileishmanial activity in our study: the inhibitors that we used to suppress their production had no effect on the ability of chitosan to kill intracellular *Leishmania* amastigotes (Fig. 3 and 5). This led us to investigate the cellular uptake of HMW chitosan and its relationship to the antileishmanial activity.

The uptake of the large charged molecule of HMW chitosan has not been systematically studied before, and there is no clear evidence of its penetration of cell membranes or of its uptake mechanism. Macrophages are known to take up extracellular materials and plasma by endocytosis. Endocytosis mainly occurs via two different cellular uptake mechanisms, pinocytosis and phagocytosis, where pinocytosis is fluid-phase endocytosis and phagocytosis is the process of engulfing large particles (56). Inhibition of pinocytosis (clathrin-mediated endocytosis [CME]) significantly reduced the antileishmanial activity of HMW chitosan. Therefore, in our study, pinocytosis (CME) was considered to be the main mechanism for the uptake of HMW chitosan by BMMs, indicating a direct antileishmanial effect of this molecule against amastigotes. Other studies have previously reported pinocytosis as the pathway for the uptake of chitosan of different molecular weights by HEK293 epithelial cells (57). The results of fluorescence imaging in our study showed that in BMMs, HMW chitosan is taken up into the parasitophorous vacuole (PV) where the *Leishmania* parasites reside, with the labeled chitosan being internalized within 4 h and increasing up to 24 h later. This is consistent with another study where rhodamine isothiocyanate-chitosan (molecular weight of 113 kDa was labelled with RITC) was found to be delivered directly to the U937 macrophage lysosome after 24 h (58). The accumulation of chitosan in the PV might be due to chitosan’s relatively high pK_a_ of 6.3 making it more soluble and protonated in the acidic contents of the vacuole. This is consistent with the results of a study using bafilomycin to inhibit acidification and prevent chitosan accumulation within macrophages (58).

In summary, our studies indicate that chitosan and its water-soluble derivatives showed antileishmanial activity against both L. major and L. mexicana promastigotes and amastigotes in a pH-dependent manner. At pH 6.5, HMW chitosan is more active than MMW and LMW chitosan and chitosan derivatives, in particular those where the amino groups are replaced. In addition, HMW chitosan activated M1 macrophages, stimulating them to produce NO and ROS. However, the antileishmanial activity of chitosan was not due to such immune activation, as an NO inhibitor and an ROS scavenger failed to reduce the antileishmanial activity. Instead, the antileishmanial activity was related to direct uptake of chitosan into the parasitophorous vacuole by pinocytosis (CME). HMW chitosan demonstrated effective *in vitro* antileishmanial activity with minimal cytotoxicity, and future work will focus on *in vivo* studies, formulations, and routes of administration.

## MATERIALS AND METHODS

### Drugs and chemicals.

Stocks of amphotericin B deoxycholate (5.2 mM [aqueous]) (Fungizone; Gibco, UK) were prepared, aliquoted, and kept at −20°C until use. Chitosan with three different molecular weights and its derivatives were used and are summarized in Table 4 and Fig. 8 (28, 59, 60, 61). Solutions of chitosan and derivatives were prepared by dissolving 1 g in 100 ml of 1% (vol/vol) acetic acid solution at room temperature with continuous stirring for 24 h until a clear solution was obtained. The pH of the solution was adjusted to approximately 6 by adding sodium hydroxide 2N (NaOH; Sigma, UK) solution and monitoring with a pH meter (Orion model 420A). The chitosan solutions were autoclaved (121°C for 15 min). Phosphorylcholine (PC)-substituted chitosan generated through reductive amination of PC-glyceraldehyde with primary amines of deacetylated chitosan (57 kDa) was kindly provided by F. Winnik (Montreal University, Canada). The percentages of substitution were controlled and determined by nuclear magnetic resonance (NMR) spectroscopy (28). Chitosan’s pK_a_ is approximately 6.3, and therefore, the approximate degrees of ionization of chitosan are 61% and 6% at pH 6.5 and 7.5, respectively.

**TABLE 4:** Details of chitosan and its derivatives used in the study

Compound (source)	Chitosan mol wt (kDa unless otherwise specified); further description	Supplier
HMW (crustacean shells)	310–375	Sigma, UK
MMW (crustacean shells)	190–310	Sigma, UK
LMW (crustacean shells)	50–190	Sigma, UK
Fungal chitosan (white mushroom)	110–150	S. Somavarapu
Chitosan oligosaccharide	≤5 Da	S. Somavarapu
Chitosan oligosaccharide-lactate	Avg Mn 5; oligosaccharide, 60%	S. Somavarapu
Chitosan-HCl	47–65	S. Somavarapu
Carboxymethyl chitosan	543.519 Da; level of substitution, 95%	S. Somavarapu
PC1-chitosan^*a*^	33; PC mol%, 30	F. Winnik
PC2-chitosan^*a*^	108; PC mol%, 20	F. Winnik
PC3-chitosan^*a*^	109; PC mol%, 30	F. Winnik

a Phosphorylcholine-substituted chitosan.

### Ethics statement.

All animal work was carried out under a UK Home Office project license according to the Animal (Scientific Procedures) Act 1986 and the new European Directive 2010/63/EU. The project license (70/8427) has been reviewed by the LSHTM Animal Welfare and Ethical Review Board prior to submission and consequent approval by the UK Home Office.

### Cell lines.

Peritoneal mouse macrophages (PEMs) were obtained from 8- to 12-week-old female CD-1 mice (Charles River Ltd., UK). Two milliliters of a 2% (wt/vol) starch solution in phosphate-buffered saline (PBS; Sigma, UK) was injected intraperitoneally. After 24 h, the animal was sacrificed and the PEMs were harvested by peritoneal lavage with cold RPMI 1640 medium (Sigma, UK) containing 200 units penicillin and 0.2 mg streptomycin per milliliter (PenStrep; Sigma, UK). Subsequently, PEMs were centrifuged at 450 × *g* at 4°C for 15 min, and then the pellet was resuspended in RPMI 1640 with 10% (vol/vol) heat-inactivated fetal calf serum (HIFCS; Gibco, UK).

Bone marrow-derived macrophages (BMMs) were obtained from femurs of 8- to 12-week-old female BALB/c mice (Charles River Ltd.). Briefly, the bone marrow cells were carefully flushed from the bone with Dulbecco’s modified Eagle’s medium (DMEM; Thermofisher, UK) with 10% (vol/vol) HIFCS, 100 U/ml penicillin, and 100 mg/ml streptomycin (Sigma, UK). Cells were pelleted by centrifugation (450 × *g* for 10 min) and resuspended in 10 ml DMEM with 10% (vol/vol) HIFCS and 50 ng/ml human macrophage colony-stimulating factor (HM-CSF; Thermofisher, UK). After plating out in T175 flasks (Greiner Bio-One, Stonehouse, UK), BMMs were kept at 37°C, 5% CO_2_ for 7 to 10 days, after which they were harvested, counted, and used.

THP-1 cells are a human leukemic monocyte-like-derived cell line. THP-1 cells were cultured in RPMI 1640 medium supplemented with l-glutamine and 10% HIFCS. THP-1 cells were incubated in RPMI 1640 plus 10% (vol/vol) HIFCS and 20 ng/ml phorbol 12-myristate 13-acetate (PMA; Sigma, UK) at 37°C and 5% CO_2_ for 72 h to induce maturation transformation of these monocytes into adherent macrophages (50).

Human squamous carcinoma (KB) cells are adherent cells derived from epidermal carcinoma in the mouth. KB cells were cultured in RPMI 1640 medium with 10% HIFCS.

The numbers of cells and macrophages were estimated by counting with a Neubauer hemocytometer by light microscopy (×400 total magnification).

### Parasites.

Four *Leishmania* strains were used, two of which (L. major [MHOM/SU/73/5ASKH] and L. mexicana [MNYC/BZ/62/M379], kindly donated by G. Getti [University of Greenwich, UK]) were GFP labeled for the fluorescence microscopy study. They were cultured in Schneider’s insect medium (Sigma, UK) with 23% (vol/vol) HIFCS, 1× penicillin-streptomycin-glutamine (Gibco-Invitrogen) and supplemented with 700 μg/ml G418 (an aminoglycoside antibiotic; Sigma, UK). L. major (MHOM/SA/85/JISH118) and L. mexicana (MNYC/BZ/62/M379) were used for other experiments without the G418. Promastigotes were incubated at 26°C, and the maximum passage number used was 7.

### *In vitro* cytotoxicity assays.

Resuspended KB cells (4 × 10^4^/100 μl) were allowed to adhere to the bottom of a 96-well plate overnight and then exposed to specific concentrations of the tested compounds for 72 h at 37°C and 5% CO_2_ in an incubator. Podophyllotoxin (Sigma, UK) was included as a positive control at a starting concentration of 0.05 μM. Cytotoxicity was evaluated by a cell viability assay using resazurin sodium salt solution (Sigma, UK), which was prepared according to the manufacturer’s instructions. Twenty microliters of the resazurin solution was added to each well of the plates, and fluorescence (cell viability) (62) was measured over a period of 1 to 24 h using a SpectraMax M3 plate reader (excitation/emission at 530/580 nm and 550-nm cutoff). Results were expressed as follows: percentage of inhibition = (100 – *x*)% viability (mean ± standard deviation [SD] σ). Cytotoxicity was evaluated in RPMI 1640 at two pHs (at the normal pH of RPMI, pH 7.5, and at a lower pH of 6.5). The pH of RPMI 1640 was reduced from 7.5 to 6.5 by adding 0.05 M acidic buffer 2-*N*-morpholino ethanesulfonic acid (MES; Sigma, UK). RPMI 1640 plus MES (0.05 M) at pH 6.5 did not show any cytotoxicity to KB cells.

### *In vitro* 72-h activity of chitosan and its derivatives against extracellular L. major *and* L. mexicana promastigotes.

Promastigotes in RPMI 1640 medium were tested while in the exponential growth phase. The promastigotes were diluted to a density of 5 × 10^6^ promastigotes/ml and then exposed to different concentrations of HMW, MMW, and LMW chitosan, chitosan derivatives, and amphotericin B (positive control) in sterile 96-well flat-bottom culture plates for 72 h at 26°C. The activity of the compounds against promastigotes was evaluated using the resazurin sodium salt solution (Sigma, UK) as described above. pH plays a critical role in the solubility and protonation of chitosan, so the activity against promastigotes was evaluated at two different pHs (pH 7.5 and a lower pH of 6.5 obtained by adding MES). The results were expressed as follows: percentage of inhibition = 100% − *x*% viability (mean ± SD).

### *In vitro* 72-h activity of chitosan and its derivatives against intracellular amastigotes of L. major and L. mexicana.

One hundred microliters of PEM culture at 4 × 10^5^ cells/ml was dispensed into each well of a 16-well LabTek tissue culture slide (Thermo Fisher, UK) at pH 7.5 or pH 6.5 and incubated for 24 h at 37°C in 5% CO_2_. After 24 h, the wells were washed with fresh culture medium to remove nonadherent cells. Stationary-phase, low-passage-number *Leishmania* promastigotes were then added to PEMs at a ratio of 5:1. This infection ratio was previously found to give sufficiently high and reproducible infection levels. Slides were incubated for another 24 h at 34°C to mimic dermal temperatures in 5% CO_2_. Any free, extracellular parasites were removed by washing the wells with cold culture medium. One slide was fixed with 100% methanol for 2 min and stained with 10% Giemsa stain for 5 min. The number of PEMs infected with *Leishmania* amastigotes per 100 macrophages was counted under a microscope. All the experiments were conducted at macrophage infection levels of above 80% prior to the addition of chitosan. Chitosan, its derivatives, and amphotericin B solutions at a range of concentrations (in quadruplicate) were added to the wells (100 μl), and the slides were incubated for 72 h at 34°C in 5% CO_2_. After 72 h, the slides were fixed with 100% methanol for 2 min and stained with 10% Giemsa stain for 5 min. The slides were examined, and the percentages of macrophages infected were determined. The antileishmanial activities of compounds were expressed as the percentages of reduction in infected macrophages compared to the numbers of infected macrophages in untreated control wells (63). RPMI 1640 plus MES (0.05 M) at pH 6.5 had no activity against *Leishmania* amastigotes.

### Influence of the origin of the host cell on the *in vitro* activity of HMW chitosan against L. major amastigotes.

A further two host cell types, THP-1 and BMMs, were infected with Leishmania major and the activity of HMW chitosan was assessed. THP-1 cells (cultured in RPMI 1640 plus 10% HIFCS) and BMMs (cultured in DMEM plus 10% HIFCS) were used to assess the host cell dependence of the antileishmanial activity of HMW chitosan (50). The experiment was conducted as described above for the assay of chitosan activity against intracellular amastigotes of L. major and L. Mexicana at pH 6.5.

### The role of HMW chitosan in BMM activation.

We chose BMMs to evaluate the activation effects of HMW chitosan and to study the cell uptake of chitosan as this macrophage population is more homogenous than those of PEMs and THP-1 cells (64); both PEMs and BMMs have been reported to have similar acidic pHs of ≈5.5 in parasitophorous vacuoles of L. amazonensis-infected cells (65–67). One hundred microliters of BMMs (4 × 10^5^/ml) in DMEM at pH 6.5 was dispensed into each well of 96-well plates (standard clear plates for nitric oxide assay and black-wall, clear-bottom plates for ROS and TNF-α assay) and incubated for 24 h at 37°C in 5% CO_2_. Plates were washed with DMEM to remove nonadherent macrophages. L. major at a 1:5 ratio (5 parasites per host cell) was then added to the wells, and the plates were incubated for 24 h at 34°C in 5% CO_2_ to allow infection of the adherent macrophages. After 24 h of incubation with macrophages, the infection rate was more than 80%. The effects of HMW chitosan on BMM activation at pH 6.5 were determined by quantifying the release of TNF-α, ROS, and NO as described below.

**(i) Measurement of TNF-α.** HMW chitosan at concentrations of 1.64, 4.9,14.8, 44.4, 133.3, and 400 μg/ml was added to infected and uninfected macrophages (see “Uptake of chitosan by macrophages” above), and the plates were incubated for 4 or 24 h at 34°C in 5% CO_2_. Lipopolysaccharides from Escherichia coli O26:B6 (LPS, 100 ng/ml; Sigma, UK) were used as a positive control and inducer. TNF-α release by the BMMs was measured using a mouse TNF-α enzyme-linked immunosorbent assay (ELISA) kit (product number ab208348; abcam, UK) according to the manufacturer’s instructions, using a SpectraMax M3 microplate reader (wavelength 450 nm).

**(ii) Measurement of ROS.** ROS was measured using a 2′,7′-dichlorofluorescein diacetate (DCFDA) cellular reactive oxygen species detection kit (abcam, UK). Uninfected and infected macrophages were treated with 25 μM DCFDA in PBS for 45 min at 37°C and then washed once in the buffer. The cells were cultured at 34°C in 5% CO_2_ for 0.5, 1, 2, 4, 8, and 24 h, with 1.64, 4.9,14.8, 44.4, 133.3, and 400 μg/ml of HMW chitosan or in the presence of H_2_O_2_ (25 mM) (Thermofisher, UK) as a positive control, in DMEM plus 10% HIFCS (pH 6.5) in quadruplicate wells. In some experiments, cells were pretreated with a selective inhibitor of ROS, *N*-acetyl-l-cysteine (NAC) (5 mM; Sigma, UK) for 2 h before the addition of the inducer or chitosan. At 0.5, 1, 2, 4, 8, and 24 h, the plates were read, using a SpectraMax M3 microplate reader (excitation/emission at 485/535 nm).

**(iii) Measurement of NO.** NO was measured using Griess reagent (Thermofisher, UK). HMW chitosan at concentrations of 1.64, 4.9,14.8, 44.4, 133.3, and 400 μg/ml was added to infected and uninfected macrophages, and the plates were incubated at 34°C in 5% CO_2_ for 4 and 24 h. LPS (100 ng/ml) was used as a positive control. In some experiments, cells were pretreated with the selective inhibitor of nitric oxide N^G^-methyl-l-arginine acetate salt (L-NMMA) (0.4 mM; Sigma, UK) for 2 h before the addition of LPS. NO was quantified according to the kit protocol. Briefly, 150 μl of the cell culture supernatants (particulates were removed by centrifugation) was mixed gently with 150 μl of the Griess reagent in a 96-well plate and the mixture was incubated for 30 min at room temperature. The absorbance was measured using a SpectraMax M3 plate reader (wavelength 548 nm). Sodium nitrite (Sigma, UK) at different concentrations was used to create a standard curve (68).

### Uptake of chitosan by macrophages.

The uptake of HMW chitosan was evaluated using two methods. The first method used two endocytosis inhibitors: cytochalasin D (1 μg/ml; Sigma, UK), which is a phagocytosis inhibitor, and dynasore (30 μg/ml; Sigma, UK), which inhibits pinocytosis (clathrin-mediated endocytosis [CME]) by blocking the GTPase activity of dynamin (69–71). The second method used dynasore and rhodamine-labeled chitosan (200 kDa; Creative PEGWorks, USA) to track cellular uptake of chitosan over time by fluorescence microscopy.

**(i) Activity of chitosan after inhibition of the endocytic pathway of BMMs.** One hundred microliters of BMM culture (4 × 10^5^/ml) in DMEM at pH 6.5 or pH 7.5 was dispensed into each well of 16-well LabTek culture slides and infected with stationary-phase L. major promastigotes. Some of the infected BMMs were pretreated with dynasore (30 μg/ml) or cytochalasin D (1 μg/ml) for 2 h. Subsequently, HMW chitosan was added to each well at concentrations of 1.64, 4.9,14.8, 44.4, 133.3, and 400 μg/ml and macrophages were incubated for 4 or 24 h at 34°C in 5% CO_2_. After each time point, the slides were examined as described above for the assay of chitosan activity against intracellular amastigotes of L. major and L. mexicana. The inhibition activity of the uptake (phagocytosis or pinocytosis) of the two inhibitors was evaluated on a fluorescence plate reader using fluorescent latex beads and pHrodo red dextran (72). We showed that cytochalasin caused 94 and 84% inhibition of phagocytosis of fluorescent latex beads (Sigma-Aldrich, UK) after 4 h and 24 h, respectively, and dynasore caused 95 and 90% inhibition of pinocytosis of pHrodo red dextran (*M*_w_ = 10,000; Thermo Fisher, UK) after 4 h and 24 h, respectively (Table S6 in the supplemental material).

**(ii) Microscopic imaging of the cellular uptake of rhodamine-labeled chitosan.** The qualitative characterization of chitosan uptake of cells was carried out by wide-field microscopy (Nikon Ti-E inverted microscope). Briefly, after deriving BMMs, 500 μl of the BMMs (in DMEM plus 10% HIFCS at pH 6.5, 4 × 10 ^4^ macrophages per ml) was seeded in each well of a 4-well LabTek tissue culture slide (Thermo Fisher, UK) and incubated for 24 h at 37°C in 5% CO_2_. Subsequently, 5 μg/ml of the nuclear dye Hoechst 33342 (excitation/emission at 350/461 nm; Thermofisher, UK) was added and the slides were incubated for 30 min at 37°C in 5% CO_2_. The macrophages were washed with PBS, and then L. major-GFP or L. mexicana-GFP parasites were added at a ratio of 10:1 and further incubated for 24 h at 34°C in 5% CO_2_ (we used a 10:1 ratio, not 5:1 as described above, as in this experiment, different strains of L. major-GFP and L. mexicana-GFP were used and the ratio 10:1 was sufficient to obtain a high infection rate). Macrophages were then washed with PBS and 500 μl of LysoTracker deep red (50 nM, excitation/emission at 647/668 nm; Thermo Fisher, UK) was added to each well. The labeled, infected macrophages were then exposed to 30 μg/ml rhodamine-labeled chitosan (200 kDa; Creative PEGWorks, USA) in 500 μl of fresh DMEM plus 10% HIFCS, pH 6.5, and incubated for 4 and 24 h at 37°C with live imaging at each time point. In some experiments, infected BMMs were preincubated with dynasore 30 μg/ml for 2 h before adding rhodamine-labeled chitosan. All the images were collected using a Nikon Ti-E inverted microscope equipped with a 63× objective using Nikon Elements software. Three images for each experiment were then analyzed using ImageJ software. The degree of correlation between pixels in the red and green channels was assessed by using the Colocalization Colormap plugin in the ImageJ software. This plugin enables quantitative visualization of colocalization by calculating the normalized mean deviation product (nMDP) in a color nMDP scale (from −1 to 1): negative indexes (cold colors) refer to no colocalization, while indexes of more than 0 (hot colors) display colocalization and higher numbers refer to greater colocalization (73, 74).

### Statistical analysis.

Dose-response curves and EC_50_s were calculated using GraphPad Prism version 7.02 software, and the corresponding sigmoidal dose-response curves were established by using a nonlinear fit with variable slope models. Results are given as mean values ± SD. EC_50_s were compared by using extra-sum-of-squares F tests. The *t* test was used to compare differences between means of two or more groups, and *P* values of 0.05 were considered statistically significant.

## Supplementary Material

Supplemental file 1
