# Supplementary material for: Activity of Chitosan and Its Derivatives against Leishmania major and Leishmania mexicana In Vitro
Source: Antimicrob Agents Chemother. 2020 Feb 21;64(3):e01772-19. doi: 10.1128/AAC.01772-19 (PMC7038302; doi:10.1128/AAC.01772-19)
Supplement: Supplemental file 1 [file AAC.01772-19-s0001.pdf]

TABLE S1 ROS production in uninfected and *L. major* infected BMMs after 8 h of exposure to different concentrations of HMW chitosan at pH=6.5

	ROS (Relative Fluorescence Intensity) in:	
**Chitosan μg/ml**	**Uninfected BMMs**	**Infected BMMs**
**1.64**	4000±100	2650±100
**4.9**	3999±200	2550±150
**14.81**	4020±150	2650±100
**44.4**	4050±100	2750±200
**133.3**	4000±200	2564±150
**400**	3959±100	2400±100
**Negative control = BMMs not exposed to chitosan**	4750±100	2850±100

Experiments were conducted in quadruplicate, data is expressed as mean +/- SD (experiment was reproduced a further two times with confirmed similar data (not shown).

TABLE S2 ROS and NO production in uninfected and *L. major* infected BMMs after exposure to chitosan in the presence of ROS scavenger and NO inhibitor at pH=6.5

	ROS (Relative Fluorescence Intensity) after 4 h in:		NO μM after 24 h in:	
**Chitosan μg/ml**	**Uninfected BMMs pre-treated with ROS scavenger**	**Infected BMMs pre-treated with ROS scavenger**	**Uninfected BMMs pre-treated with NO inhibitor**	**Infected BMMs pre-treated with NO inhibitor**
**1.64**	4700 ±200	2850±150	1.4± 0.4	0.15± 0.1
**4.9**	4800±250	2750±200	1.5± 0.3	0.16± 0.1
**14.81**	4750±100	2950±150	1.9± 0.5	0.17± 0.1
**44.4**	4800±100	2750±100	1.6± 0.2	0.15± 0.1
**133.3**	4900±150	2864±100	1.2± 0.4	0.14± 0.1
**400**	4950±100	2600±100	1.0± 0.6	0.15± 0.1
**Positive control (ROS) = BMMs treated with 25 mM H_2O_2**	4800±250	2750±100		
**Positive control (NO) = BMMs treated with 10 μg/ml LPS**			1.4± 0.3	0.16± 0.1
**Negative control = BMMs not exposed to**	4800±100	2900±100	1.7± 0.3	0.13± 0.1

chitosan or to LPS or H_2O_2				

Experiments were conducted in quadruplicate, data is expressed as mean +/- SD (experiment was reproduced a further two times with confirmed similar data (not shown). ROS was measured after 4 h and NO was measured after 24 h of exposure to HMW chitosan.

TABLE S3 NO production in uninfected and *L. major*-infected BMMs after 4h of exposure to different concentrations of HMW chitosan at pH=6.5

	NO production (uM) in:	
**Chitosan µg/ml**	**Uninfected BMMs**	**Infected BMMs**
**1.64**	0	0
**4.9**	0	0
**14.81**	0	0
**44.4**	0.05±0.01	0
**133.3**	0.06±0.01	0.05±0.01
**400**	0.05±0.01	0.04±0.01
**Negative control = BMMs not exposed to chitosan**	0.07±0.01	0.05±0.01

Experiments were conducted in quadruplicate, data is expressed as mean +/- SD (experiment was reproduced a further two times with confirmed similar data (not shown).

TABLE S4 In *vitro* activity of chitosans against promastigotes based on molarity

**Compound**	**pH=6.5*,*** ***L. major***		**pH=6.5*,*** ***L. mexicana***	
	**EC_{50} µM**	**EC_{90} µg/ml**	**EC_{50} µM**	**EC_{90} µg/ml**
**HMW chitosan**	0.017± 0.001	0.10± 0.02	0.03± 0.005	0.28± 0.1
**MMW chitosan**	0.024± 0.001	0.172± 0.03	0.04± 0.005	0.38± 0.1
**LMW chitosan**	0.05± 0.001	0.33± 0.06	0.085± 0.005	0.7± 0.2
**Fungal chitosan**	0.05± 0.003	0.31± 0.005	0.08± 0.01	0.5± 0.1

Experiments were conducted in triplicate, data is expressed as mean +/- SD (experiment was reproduced a further two times with confirmed similar data (not shown).

TABLE S5 In *vitro* activity of chitosans against amastigotes based on molarity

**Compound**	**pH 6.5*** ***L. major***		**pH 6.5*** ***L. mexicana***	
	**EC_{50} µM**	**EC_{90} µM**	**EC_{50} µM**	**EC_{90} µM**
**HMW chitosan**	0.03± 0.01	0.2± 0.05	0.044±0.005	0.3± 0.08
**MMW chitosan**	0.05± 0.04	0.32± 0.07	0.06±0.008	0.5± 0.1
**LMW chitosan**	0. 1± 0.008	0.6± 0.1	0.13±0.01	0.97± 0.3
**Fungal chitosan**	0.09±0.002	0.7± 0.2	0.13 ±0.01	1.1± 0.3

Experiments were conducted in quadruplicate, data is expressed as mean +/- SD (experiment was reproduced a further two times with confirmed similar data (not shown).

TABLE S6 Phagocytosis and pinocytosis by *L. major* infected BMMs in the presence of the uptake inhibitors

	**Number of latex beads ± SD *10^5/mg protein**		**Concentration of dextran ± SD µg/mg protein**	
**Time/Hour**	**Without cytochalasin D**	**With cytochalasin D**	**Without dynasore**	**With dynasore**
**4**	108 ± 8	6 ± 1	4.9 ± 0.5	0.2 ± 0.1
**24**	456 ± 30	73 ± 8	18.9 ± 1	1.8 ± 0.2

Experiments were conducted in triplicate, data is expressed as mean +/- SD (experiment was reproduced a further two times with confirmed similar data (not shown).

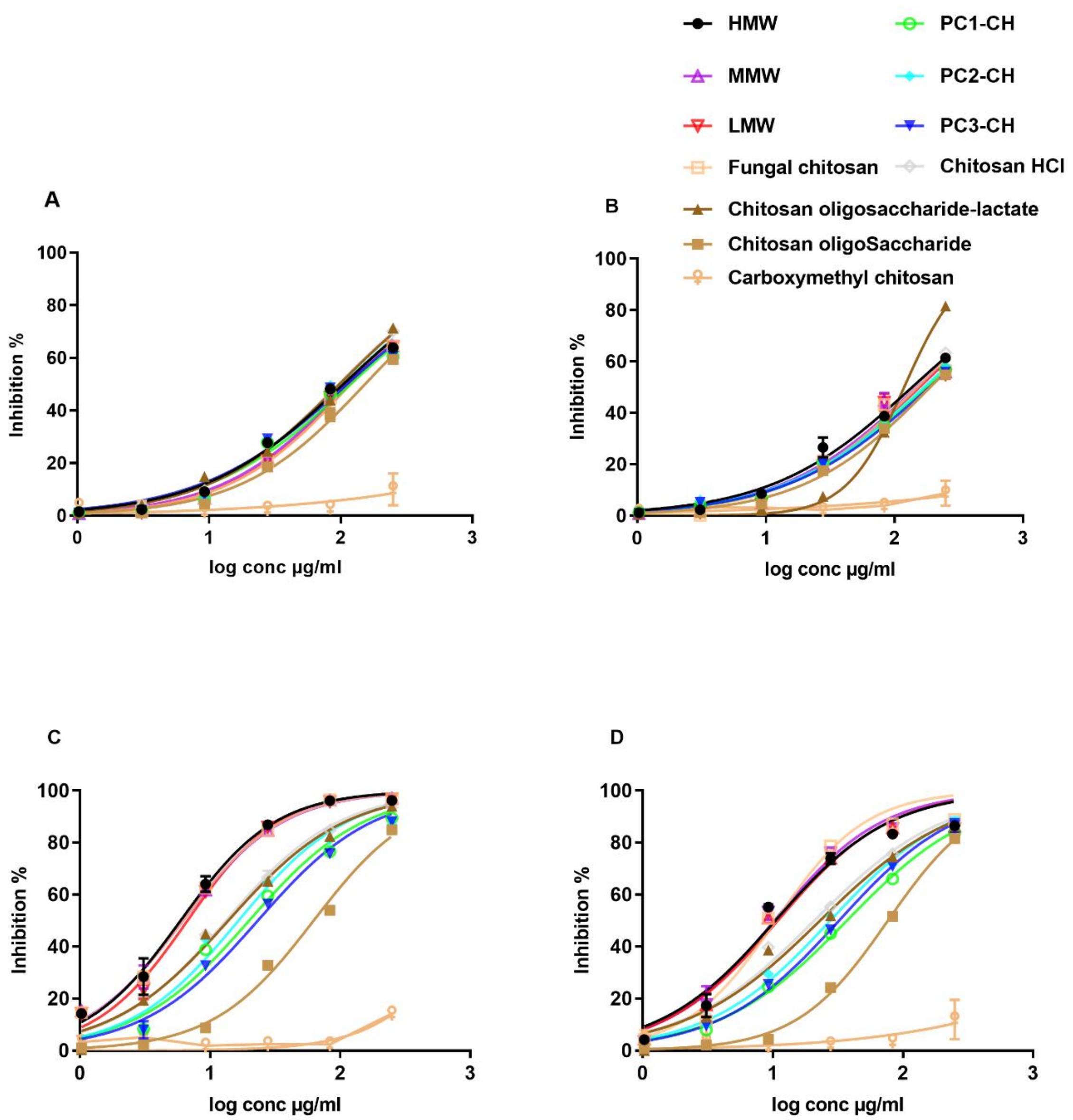

Fig S1 Dose response curves of the activity of chitosan and its derivatives against *Leishmania* promastigotes at two pH values. A: *L. major* at pH=7.5; B: *L. mexicana* at pH = 7.5; C: *L. major* at pH = 6.5; D: *L. mexicana* at pH = 6.5. Promastigotes were cultured in the presence of different concentrations of chitosan and its derivatives. The activity of drugs was measured after 72h by the resazurin solution. Values are expressed as % inhibition of promastigotes relative to untreated controls. Statistically significant difference was observed in EC_{50} values of chitosan and its derivatives against *L. mexicana* and *L. major* promastigotes between pH=6.5 and pH=7.5 ($p<0.05$ by t-test).

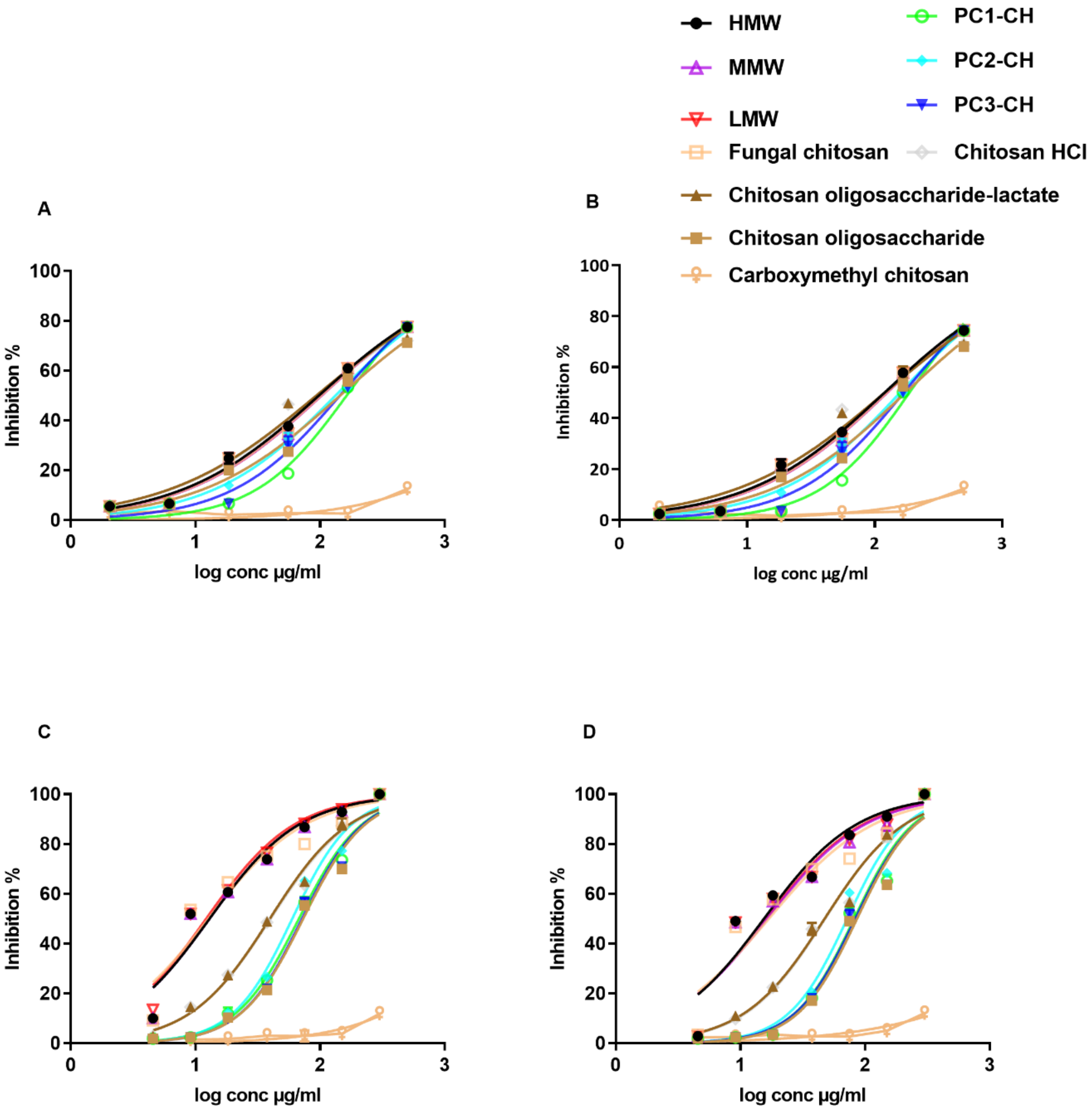

Fig S2 Dose response curves of the activity of chitosan and its derivatives against *Leishmania* amastigotes at two pH values. A: *L. major* at pH=7.5; B: *L. mexicana* at pH = 7.5; C: *L. major* at pH = 6.5; D: *L. mexicana* at pH = 6.5. PEMs were infected with stationary-phase promastigotes and exposed to various concentrations of chitosan and its derivatives, followed by microscopic counting of the number of infected macrophages*. Values are expressed as % inhibition of infection relative to untreated controls. Chitosan and its derivatives are significantly more active in pH 6.5 than in pH 7.5 ($p<0.05$ by t-test). * Macrophage infection rate was >80% after 24h.

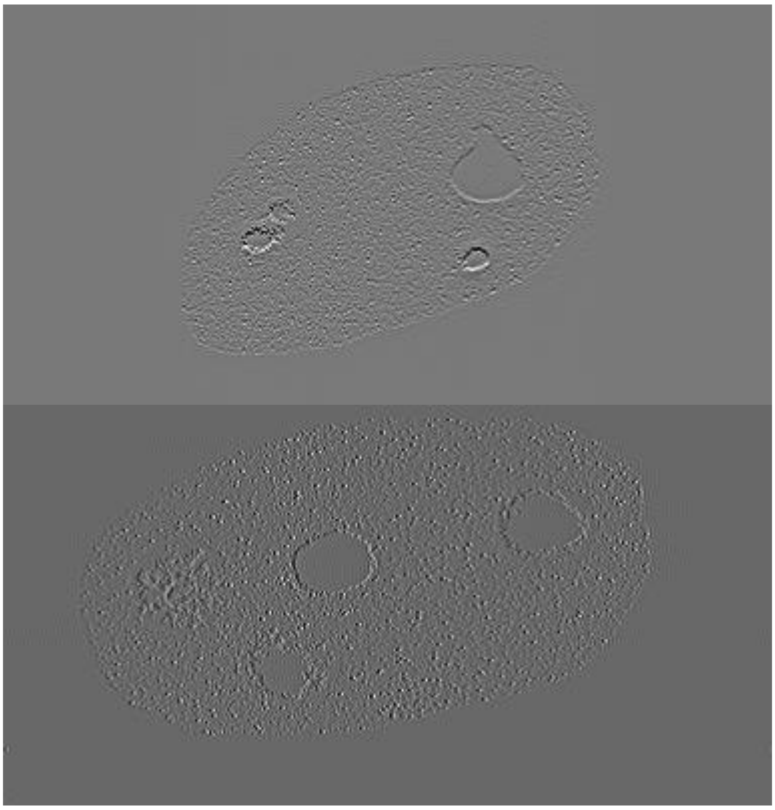

FIG S3. Confocal Phase images of BMMs infected with *L. major* (A) and *L. mexicana* (B) were analysed by the ImageJ software in correlation with figure 7 in the manuscript. 63x magnification
